# Animal Models and Their Role in Imaging-Assisted Co-Clinical Trials

**DOI:** 10.3390/tomography9020053

**Published:** 2023-03-16

**Authors:** Donna M. Peehl, Cristian T. Badea, Thomas L. Chenevert, Heike E. Daldrup-Link, Li Ding, Lacey E. Dobrolecki, A. McGarry Houghton, Paul E. Kinahan, John Kurhanewicz, Michael T. Lewis, Shunqiang Li, Gary D. Luker, Cynthia X. Ma, H. Charles Manning, Yvonne M. Mowery, Peter J. O’Dwyer, Robia G. Pautler, Mark A. Rosen, Raheleh Roudi, Brian D. Ross, Kooresh I. Shoghi, Renuka Sriram, Moshe Talpaz, Richard L. Wahl, Rong Zhou

**Affiliations:** 1 Department of Radiology and Biomedical Imaging, University of California San Francisco, San Francisco, CA 94158, USA; john.kurhanewicz@ucsf.edu (J.K.); renuka.sriram@ucsf.edu (R.S.); 2 Department of Radiology, Duke University Medical Center, Durham, NC 27710, USA; cristian.badea@duke.edu; 3 Department of Radiology and the Center for Molecular Imaging, University of Michigan School of Medicine, Ann Arbor, MI 48109, USA; tlchenev@umich.edu (T.L.C.); gluker@umich.edu (G.D.L.); bdross@umich.edu (B.D.R.); 4 Molecular Imaging Program at Stanford (MIPS), Department of Radiology, Stanford University, Stanford, CA 94305, USA; heiked@stanford.edu (H.E.D.-L.); roudi@stanford.edu (R.R.); 5 Department of Medicine, Washington University School of Medicine, St. Louis, MO 63110, USA; lding@genome.wustl.edu (L.D.); shunqiangli@wustl.edu (S.L.); cynthiaxma@wustl.edu (C.X.M.); 6 Advanced Technology Cores, Baylor College of Medicine, Houston, TX 77030, USA; dobrolec@bcm.edu; 7 Fred Hutchinson Cancer Center, Seattle, WA 98109, USA; houghton@fredhutch.org; 8 Department of Radiology, University of Washington, Seattle, WA 98105, USA; kinahan@uw.edu; 9 Departments of Molecular and Cellular Biology and Radiology, Baylor College of Medicine, Houston, TX 77030, USA; mtlewis@bcm.edu; 10 Department of Microbiology and Immunology, University of Michigan School of Medicine, Ann Arbor, MI 48109, USA; 11 Department of Cancer Systems Imaging, The University of Texas MD Anderson Cancer Center, Houston, TX 77030, USA; hcmanning@mdanderson.org; 12 Department of Radiation Oncology, Duke University School of Medicine, Durham, NC 27708, USA; yvonne.mowery@duke.edu; 13 Department of Head and Neck Surgery & Communication Sciences, Duke University School of Medicine, Durham, NC 27708, USA; 14 Abramson Cancer Center, University of Pennsylvania, Philadelphia, PA 19104, USA; peter.odwyer@uphs.upenn.edu (P.J.O.); mark.rosen@pennmedicine.upenn.edu (M.A.R.); rongzhou@upenn.edu (R.Z.); 15 Department of Integrative Physiology, Baylor College of Medicine, Houston, TX 77030, USA; rpautler@bcm.edu; 16 Department of Radiology, University of Pennsylvania, Philadelphia, PA 19104, USA; 17 Department of Biological Chemistry, University of Michigan School of Medicine, Ann Arbor, MI 48109, USA; 18 Mallinckrodt Institute of Radiology (MIR), Washington University School of Medicine, St. Louis, MO 63110, USA; shoghik@wustl.edu (K.I.S.); rwahl@wustl.edu (R.L.W.); 19 Division of Hematology/Oncology, University of Michigan School of Medicine, Ann Arbor, MI 48109, USA; mtalpaz@med.umich.edu; 20 Department of Internal Medicine, University of Michigan School of Medicine, Ann Arbor, MI 48109, USA

**Keywords:** co-clinical trials, animal models, imaging, prostate cancer, sarcoma, colorectal cancer, osteosarcoma, pancreatic cancer, myelofibrosis, breast cancer, lung cancer

## Abstract

The availability of high-fidelity animal models for oncology research has grown enormously in recent years, enabling preclinical studies relevant to prevention, diagnosis, and treatment of cancer to be undertaken. This has led to increased opportunities to conduct co-clinical trials, which are studies on patients that are carried out parallel to or sequentially with animal models of cancer that mirror the biology of the patients’ tumors. Patient-derived xenografts (PDX) and genetically engineered mouse models (GEMM) are considered to be the models that best represent human disease and have high translational value. Notably, one element of co-clinical trials that still needs significant optimization is quantitative imaging. The National Cancer Institute has organized a Co-Clinical Imaging Resource Program (CIRP) network to establish best practices for co-clinical imaging and to optimize translational quantitative imaging methodologies. This overview describes the ten co-clinical trials of investigators from eleven institutions who are currently supported by the CIRP initiative and are members of the Animal Models and Co-clinical Trials (AMCT) Working Group. Each team describes their corresponding clinical trial, type of cancer targeted, rationale for choice of animal models, therapy, and imaging modalities. The strengths and weaknesses of the co-clinical trial design and the challenges encountered are considered. The rich research resources generated by the members of the AMCT Working Group will benefit the broad research community and improve the quality and translational impact of imaging in co-clinical trials.

## 1. Introduction

The Co-clinical Imaging Research Resource Program (CIRP) of the National Cancer Institute (NCI) focuses on the optimization and dissemination of quantitative imaging methods and protocols employing genetically engineered mouse models (GEMM) and patient-derived xenografts (PDX) to improve co-clinical precision medicine research [1]. The CIRP network includes ten research resources from nine teams at Washington University St. Louis, Duke University, University of Texas’s MD Anderson Cancer Center (MDACC) (originally at Vanderbilt University), University of Pennsylvania (Penn), Baylor College of Medicine/University of Texas Austin (UT Austin)/Stanford University, University of Michigan, University of California, San Francisco (UCSF), Stanford University, and University of Washington/Fred Hutchinson Cancer Center. Investigators from each team participate in working groups (WGs)—Animal Models and Co-clinical Trials (AMCT), Imaging Acquisition and Data Process (IADP), and Informatics and Outreach (IMOR)—to address common issues across the board concerning quantitative imaging. The mission of the AMCT WG is to optimize murine models of cancer to mimic the biology and response to treatment of human malignancies. The group aims to reach a consensus regarding best practices for employing animal models in co-clinical trials with preclinical quantitative imaging and to generate resources that facilitate successful implementation of reproducible animal models in co-clinical studies. The AMCT WG considers the current practices, strengths, and challenges of animal models used for co-clinical studies versus human clinical trials.

The members of the AMCT WG possess a wealth of experience in applying a multitude of imaging technologies to diverse animal models treated using a variety of therapies. Cancers targeted include triple-negative breast cancer, estrogen receptor-positive breast cancer, small cell neuroendocrine prostate cancer, osteosarcomas, high-risk localized soft tissue sarcoma of the extremity, colorectal cancer, pancreatic ductal adenocarcinoma, and non-small cell lung cancer and myelofibrosis. Murine hosts of PDX include SCID/beige, athymic nude, and NOD scid gamma (NSG) immunocompromised mice, and immunocompetent Balb/c, C57BL/6J and 129/SvJae mice are used for GEMM and murine tumors. Sites of tumors targeted for therapy and imaging include the orthotopic mammary fat pad, bone and muscle, and metastatic sites of the bone and liver. The biological fidelity of models has been confirmed by characteristics such as histologic appearance, genomics, transcriptomics, proteomics, metabolomics, and clinical progression (i.e., development of metastases). Treatments utilized in the co-clinical trials range from standard chemo- and immuno-therapies (platinum-based drugs, methotrexate, anthracyclines, taxanes, anti-PD-1 or PD-L1 antibodies), targeted therapies (anti-EGFR antibody panitumumab), hormonal therapy, surgery, and radiotherapy, to a novel CD47 monoclonal antibody, glutaminase inhibition, the JAK2 inhibitor ruxolitinib, a vitamin D receptor ligand, the CDK4/6 inhibitor abemaciclib, and an inhibitor of CXCL1/2. Endpoints selected for the co-clinical trial include tumor growth inhibition, local recurrence-free survival, metastasis-free survival, and disease-free survival. Imaging technologies include ultrasound, micro-computed tomography (CT), positron emission tomography (PET), magnetic resonance imaging (MRI), and hyperpolarized ^13^C MRI.

An overview of the co-clinical studies in the CIRP network is provided in Table 1. In the following sections, members of the CIRP AMCT WG describe their approaches to conducting co-clinical studies incorporating quantitative imaging. The clinical trial and endpoints, rationale for selection of animal model, selected therapy, imaging modalities, and strengths and weaknesses of the co-clinical trial design are considered by each member.

## 2. Co-Clinical Trials

### 2.1. The UCSF Co-Clinical Quantitative Imaging of Small Cell Neuroendocrine Prostate Cancer Using Hyperpolarized ^13^C MRI

The co-clinical study at UCSF focuses on small cell neuroendocrine prostate cancer (SCNC), a lethal variant of metastatic castration-resistant prostate cancer (mCRPC) that is becoming increasingly prevalent in the era of treatment with second-generation androgen signaling inhibitors (ASI) [2]. While lymph nodes are the most common sites for metastases, bone and liver metastases are more lethal. At these sites, SCNC may exist alone or admixed with the more common adenocarcinoma type of mCRPC. The project uses three PDX—LuCaP 93, LTL352, and LTL610—with genetic, transcriptomic, and immunohistologic features characteristic of SCNC [3,4]. These PDX are propagated in the tibia and liver of male NSG mice to mimic the microenvironment of bone and liver metastases.

The corresponding clinical study at UCSF (ClinicalTrials.gov ID: NCT04346225) aims to assess the response of SCNC to standard-of-care carboplatin chemotherapy using hyperpolarized (HP) [1-^13^C]pyruvate MRI. Patients who have mCRPC and prior progression on ASI undergo standard-of-care cross-sectional imaging of the abdomen/pelvis using either CT or MRI to guide the selection of a target lesion. Following baseline HP ^13^C MRI, patients undergo paired CT image-guided percutaneous core needle biopsies of metastases within 14 days to confirm the presence of SCNC and are given carboplatin. Consistent with standard clinical practice, the patients undergo re-staging with cross-sectional imaging of the abdomen/pelvis every 8 weeks thereafter to determine tumor response (defined as >30% reduction in longest diameter of target lesion). Patients undergo follow-up HP ^13^C MRI after 1 cycle of treatment (carboplatin area under the curve (AUC) 5 through intravenous (IV) line every 3 weeks) to investigate the reduction in the HP ^13^C MRI metric (apparent rate of conversion of [1-^13^C]pyruvate to [1-^13^C]lactate, k_PL_) as an early marker of response to chemotherapy. The multiparametric (mp) ^1^H MRI/dynamic HP ^13^C MRI protocol used includes T_1_- and T_2_-weighted anatomic, diffusion weighted, and dynamic contrast-enhanced (DCE) imaging and 2D dynamic HP [1-^13^C]pyruvate MRI for both clinical and preclinical protocols.

**Figure 1:**
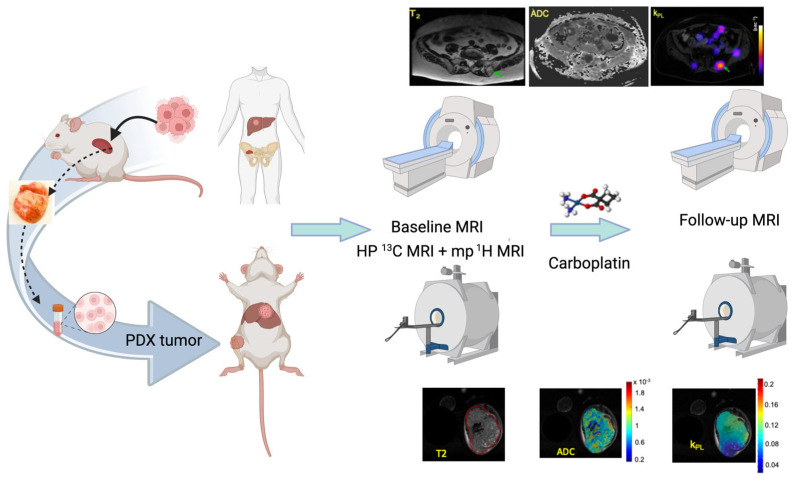
The UCSF co-clinical project schema. The project focuses on assessing the response of SCNC to standard-of-care platinum-based chemotherapy using HP [1-^13^C]pyruvate MRI. The co-clinical project uses three established SCNC PDX that are propagated in the murine kidney, digested into single cells, and inoculated in the murine tibia and liver to match the metastatic SCNC patient population under study in the clinical trial. Upon reaching a volume of ~0.3 cc, assessed by MRI, tumors are characterized by baseline dynamic HP [1-^13^C]pyruvate MRI and mp-MRI. Following one cycle of treatment with carboplatin or placebo, tumors are again evaluated by combined dynamic HP [1-^13^C]pyruvate MRI and mp-MRI to investigate the reduction in the HP ^13^C MRI metric (apparent rate of conversion of [1-^13^C]pyruvate to [1-^13^C]lactate, k_PL_) as an early marker of response to chemotherapy. As a part of both clinical and pre-clinical protocols, k_PL_ maps are overlaid on the corresponding T_2_ weighted anatomic images and correlated with changes in ADC images and tumor growth rate with treatment.

The co-clinical trial closely mimics the clinical trial, with baseline imaging and subsequent post-therapy imaging after one cycle (Figure 1). In patients, administration of carboplatin is based on the AUC of concentration-versus-time [5]. Consequently, the dose used in the clinical trial of AUC 5 through IV every 3 weeks corresponds to a maximum dose of 75 mg of carboplatin. Operating under the same constraint of a similar AUC and extrapolating from the LD_10_ in mice of 495 mg/m^2^ translates to 9 mg maximum dose/week for mice [6]. Considering the differences in tumor growth rate between men (doubling time of ~90 days) [7] and mice (~20 days in the PDX), the dose would be ~66 mg/kg/week for the co-clinical study. Based on preliminary studies with the PDX, this dose should yield a significant difference in tumor growth rate between control and treated groups and is well within the range of doses found in the literature (40–90 mg/kg/week) for murine studies [8]. Neither the clinical nor preclinical trial includes relapse/recurrence after treatment as an outcome variable.

The preclinical trial provides several advantages compared to the clinical study, including a vehicle-treated control, the standardization of the initial volume of tumors, and the ability to image mice more frequently than patients to quantitatively measure tumor response over time. Importantly, the study permits the reverse engineering of clinical acquisition and processing protocols for the development and optimization of associated preclinical protocols. The SCNC PDX have a more homogeneous phenotype compared to the mixed adenocarcinoma/SCNC and/or other variants often observed in mCRPC patients, which may be advantageous for preclinical studies but is not completely representative of the clinical presentation. The implantation of PDX cells into the bone or liver does not capture the metastatic process but does enable more clinically relevant studies on the treatment of SCNC than models involving the implantation of non-human cells or immortalized cell lines at sites that have significantly different microenvironments, such as the murine flank. Organ-specific prostate cancer responses have been observed in both clinical and preclinical studies [9,10,11,12], emphasizing the importance of the site of implantation in co-clinical studies. The absence of an immune microenvironment in NSG mice could impact both therapeutic response and imaging parameters. Cardiac and respiratory motion has not been an issue in studying SCNC bone metastases. However, respiratory motion can be an issue for studies on liver metastases. We are currently testing a very fast 2D Dynamic HP ^13^C Spectral–Spatial (SPSP) EPI acquisition used clinically and comparing it with standard 2D Dynamic HP ^13^C chemical shift imaging to evaluate the impact of motion/respiration in test–retest studies.

### 2.2. The Duke Preclinical Research Resources for Quantitative Imaging Biomarkers

The Duke co-clinical study mirrors an ongoing multi-institutional, randomized phase II clinical trial (ClinicalTrials.gov ID: NCT03092323) investigating whether the addition of neoadjuvant and adjuvant pembrolizumab, a monoclonal antibody targeting programmed cell death protein-1 (PD-1), to preoperative radiotherapy (RT) and surgical resection improves disease-free survival compared to neoadjuvant RT (50 Gy in 25 fractions) and surgery for patients with high-risk soft tissue sarcoma of the extremity (undifferentiated pleomorphic sarcoma [UPS] or dedifferentiated/pleomorphic liposarcoma [LPS]) [13]. Clinical and preclinical imaging to evaluate tumor response and monitor for metastatic disease involve MRI of the primary sarcoma and serial chest CT to evaluate for lung metastases.

**Figure 2:**
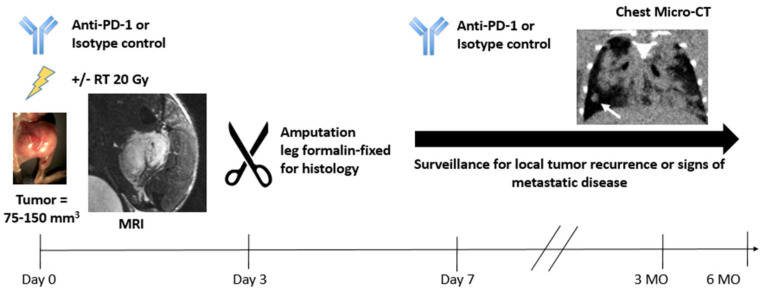
Examples of MRI and micro-CT images in the p53/MCA model and the schematics of the preclinical arm of the clinical trial.

To mimic gradual sarcoma development under immune surveillance and the frequent development of lung metastases in sarcoma patients, the preclinical arm of the co-clinical trial utilized a carcinogen-induced GEMM of sarcoma [14] that develops spontaneous lung metastases in immunocompetent mice (Figure 2). To recapitulate the genetic complexity, variable mutational load, and frequent mutation of *TP53* observed in human UPS and dedifferentiated/pleomorphic LPS [15], primary sarcomas were generated in wild-type 129/SvJae mice aged 6 to 10 weeks through the injection of adenovirus-expressing Cas9 endonuclease and sgRNA-targeting *Trp53*, followed by injection of 3-methylcholanthrene (MCA) into the gastrocnemius muscle (p53/MCA model). Tumors (detected by palpation) developed in the hind limb 7–12 weeks after induction. Primary p53/MCA sarcomas resemble human UPS in both histologic appearance and gene expression profile [16]. Without treatment, mice were euthanized within 3 weeks of tumor detection due to rapid tumor growth. To allow time for metastatic disease development, amputation of the tumor-bearing hind limb achieved primary tumor control.

Sarcoma-bearing mice were randomized into four treatment groups when tumors reached 75–125 mm^3^: isotype control antibody (ISO), anti-PD-1 antibody, ISO + 20 Gy (ISO + RT), and anti-PD-1 + 20 Gy (anti-PD-1 + RT). Anti-PD-1 or the isotype control antibody (200 μg) was administered by intraperitoneal injection on Day 0 (pre-amputation), Day 7 (day of amputation), and Day 14 (post-amputation). Sarcomas were treated with sham RT or 20 Gy (single fraction) on Day 0. Due to rapid p53/MCA tumor growth, the fractionated 5-week RT regimen and the every 3-week dosing of 200 mg pembrolizumab utilized in the clinical trial was not feasible. Primary sarcomas were imaged using micro-MRI, and respiratory-gated micro-CT was used to detect lung metastases with high spatial resolution [17]. Compared to surgery with isotype control, the combination of anti-PD-1, RT, and surgery improved local recurrence-free survival and disease-free survival, but not metastasis-free survival [18]. Mice treated with RT and surgery, but not anti-PD-1 and surgery, showed significantly improved local recurrence-free survival and metastasis-free survival over surgery alone. A challenge was the low rate of metastases (~12%) in mice treated with isotype control and surgery, leading to an underpowered study for the metastasis-free survival endpoint despite > 60 mice/group. To address this, we are evaluating alternative high-mutational load primary sarcoma models with greater propensity for lung metastases for a follow-up study.

For patients enrolled in the clinical trial SU2C-SARC032, pre-treatment tumor samples are undergoing both whole exome sequencing (with matched normal tissue) and RNA sequencing. The surgical resection specimen obtained after neoadjuvant radiation (with or without neoadjuvant anti-PD-1 therapy) is also undergoing RNA sequencing. While some patients have developed new metastases after undergoing sequencing, these results remain blinded. For the murine co-clinical trial, a subset of sarcoma samples obtained from hind limb amputation after neoadjuvant radiation with or without neoadjuvant anti-PD-1 therapy underwent whole exome sequencing. The mice received one additional dose of anti-PD-1 antibody or isotype control after amputation. Tumor samples were taken from mice who developed local recurrences after amputation of the tumor-bearing hind limb, and the recurrent tumor also underwent whole exome sequencing. These mice did not develop new metastases after genetic sequencing.

### 2.3. The MDACC PET Imaging Resource to Enhance Delivery of Individualized Cancer Therapeutics (PREDICT) for Wild-Type KRAS Colorectal Cancer

The co-clinical study at MDACC utilizes PET imaging and radiotracers of glutamine metabolism to assess the response of individualized cancer therapeutics for wild-type (WT) *KRAS* colorectal cancer (CRC) patients.

Approximately 50% of CRC are known to have a mutated *KRAS* gene, indicating that the remaining 50% of CRC patients might respond to anti-epidermal growth factor receptor (EGFR)-targeted therapy [19,20]. However, 40–60% of WT *KRAS* tumors with or without BRAF mutations, which are usually mutually exclusive with KRAS mutations, represent 5–15% of advanced CRC [21] and do not respond to such therapy [20]. Even though BRAF mutations may confer resistance to anti-EGFR therapy, the role of KRAS and BRAF mutations in CRC survival and response to standard chemotherapy regimens remains inconclusive [22,23]. To overcome the unmet needs, methods to guide the selection of the appropriate treatment for patients including treatment with EGFR monoclonal antibodies (e.g., mAbs; cetuximab and panitumumab) and novel therapeutic cocktails are needed.

The mitogen-activated protein kinase (MAPK) pathway, one of the most frequently deregulated signaling cascades in CRC, follows the ligand-mediated activation of EGFR and requires glutamine; furthermore, glutamine can induce MAPK-mediated proliferation in an EGFR-independent manner [24,25]. Indeed, we evaluated the improved therapeutic efficacy of combined blockade of EGFR and glutamine metabolism in preclinical CRC models [25]. We also demonstrated the superiority of PET imaging with radiotracers of glutamine metabolism for monitoring tumors rather than utilizing ^18^F-fluorodeoxyglucose (FDG) PET preclinically [26] and clinically [27,28].

Currently, we are conducting two clinical trials using PET imaging with ^11^C-Glutamine and (S)-4-(3-^18^F-Fluoropropyl)-L-glutamic acid (^18^F-FSPG) in WT *KRAS* CRC. In collaboration with the Vanderbilt University Medical Center, we are conducting a Phase II clinical trial combining an anti-EGFR antibody, panitumumab, with a glutaminase (GLS1) inhibitor, CB-839 (ClinicalTrials.gov ID: NCT03263429). Patients participating in this trial receive glutaminase inhibitor CB-839 orally twice a day (PO BID [29]) on Days 1–28, panitumumab IV over 60–90 min on Days 1 and 15, and irinotecan hydrochloride IV over 90 min on Day 1 and 15 (Phase I only). The treatment course repeats every 28 days in the absence of disease progression or unacceptable toxicity. The tumor lesions are monitored by ^11^C-Glutamine and ^18^F-FSPG PET/CT imaging pre-treatment and up to 8 weeks post-treatment. The baseline and post-treatment PET imaging provides a measure of glutamine avidity of the tumors and is evaluated as a predictor of treatment response. Additionally, the imaging data will be correlated to genetic (RNA-seq) and immunohistochemistry (IHC) data. In the second study, a Phase I clinical trial, baseline glutamine PET imaging with ^11^C-Glutamine and ^18^F-FSPG is evaluated in patients with metastatic WT *KRAS* CRC undergoing treatment with EGFR-targeted antibody therapy (ClinicalTrials.gov ID: NCT03275974). The goal is to identify a predictive imaging biomarker of response which would guide therapy selection for patients.

**Figure 3:**
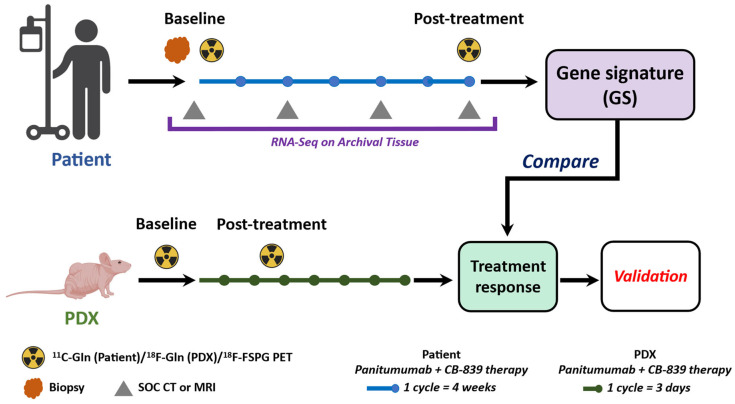
Scheme of the co-clinical trial. Patients with WT *KRAS* CRC and immunocompromised nude mice bearing WT *KRAS* CRC PDX tissues receive the therapy with combined panitumumab and CB-839. Tumors are imaged pre- and post-treatment with ^11^C-glutamine/^18^F-glutamine and ^18^F-FSPG. Complementary to genomic approaches, a PET imaging-derived gene signature associated with treatment response is established.

The preclinical trial uses avatar PDX athymic nude mouse models (Figure 3). Gene sequencing is conducted in both patients and PDX prior to treatment. The mice are treated with either vehicle or a combined regimen of CB-839 (200 mg/kg PO BID [29]) and panitumumab (40 mg/kg every 72 h [30,31]) for 3 weeks, similar to the Phase I/II clinical trial. Tumor volumes are measured manually by calipers every third day, and treatment continues until either complete regression or tumor volume exceeds the Institutional Guidelines for mouse health (1000-mm^3^), at which point mice will be sacrificed. The preclinical PET/CT scans with ^18^F-4-fluoro-glutamine and ^18^F-FSPG are conducted pre- and 1-week post-treatment. We are also using RNA-seq data from these PDX to develop imaging-derived gene signatures associated with treatment response. These gene signatures could provide rationale and guidance for the appropriate treatment selection with combined blockade of EGFR and glutamine metabolism.

### 2.4. Stanford University Co-Clinical Research for Imaging Tumor-Associated Macrophages

The overall goal of the project at Stanford University is to optimize and validate preclinical and clinical quantitative imaging techniques for in vivo quantification of tumor associated macrophages (TAM) in osteosarcomas. This goal will be accomplished by optimizing and validating preclinical quantitative imaging methods for TAM imaging, implementing the optimized methods in a co-clinical trial, and populating a web-accessible research resource.

While the outcome of patients with localized bone sarcomas has significantly improved over the last two decades, the overall survival of patients with metastatic disease continues to be less than 30% [32,33,34,35,36,37]. Hence, new therapeutic targets are desperately needed for patients with bone sarcomas. Important predictors of outcome for newly diagnosed bone sarcomas include patient age, tumor size, location, grade/histology, and stage [38]. However, these parameters do not define specific biologic tumor characteristics which could serve as targets for individualized therapies. TAMs have been recognized as an independent predictor of tumor prognosis and a powerful target for novel immuno-therapies. CD47 is a surface molecule on cancer cells that functions as a “don’t eat me” signal for TAM by engaging signal-regulatory protein alpha (SIRPα), an inhibitory receptor on macrophages [39,40]. CD47 mAbs inhibit the interaction between CD47 and SIRPα and thereby activate TAM to phagocytose cancer cells [41,42,43,44,45]. Combining TAM-activating immunotherapies with clinical standard chemotherapy in patients with osteosarcomas is difficult because there are no clinically established biomarkers that can monitor TAM responses in vivo. The Stanford team have developed a clinically available TAM imaging test that relies on intravenous injection of the FDA-approved iron supplement ferumoxytol (Feraheme^TM^) [46,47,48,49]. Ferumoxytol is composed of nanoparticles which are phagocytosed by TAM and can be detected by MRI [50,51]. The purpose of this study is to optimize and validate this preclinical and clinical quantitative imaging technique for in vivo quantification of TAM in osteosarcomas.

**Figure 4:**
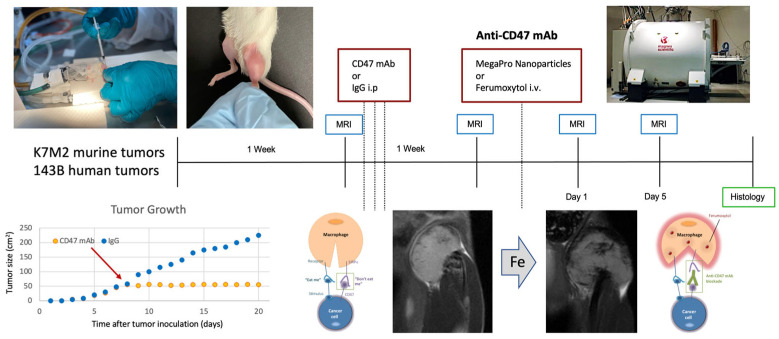
The Stanford co-clinical trial design.

The Stanford co-clinical trial design is outlined in Figure 4. The team investigates mice with orthotopically implanted murine or human osteosarcomas and treats them with CD47 mAb or IgG1 control antibody. The expression of CD47 was assessed in de-identified osteosarcoma specimens from eight chemotherapy-naïve human patients, one de-identified osteoma specimen, one de-identified normal bone specimen (Cooperative Human Tissue Network), three human osteosarcoma cell lines (U-2 OS, Saos-2, and MNNG/HOS) and a normal bone cell line (hFOB 1.19) by qPCR, using glyceraldehyde 3-phosphate dehydrogenase (GAPDH) as a control marker [50].

Animals undergo MRI scans before and after treatment as well as before and after undergoing intravenous infusion of ferumoxytol or Mega Pro iron oxide nanoparticles. Doses of CD47 mAb and iron oxide nanoparticle are adjusted according to the weight of the mouse or patient [50,51]. Ferumoxytol doses in mice are 30 mg Fe/kg, whereas in human patients, doses are 5 mg Fe/kg. Mice receive a higher dose due to faster biodistribution. Mice bearing murine tumors are treated with CD47 mAb (clone MIAP301) at a dose of 10 mg/kg and mice bearing human tumors are treated with CD47 mAb (clone B6H12) at a dose of 10 mg/kg on Days 1, 3, and 5. MRI is performed at various time points after treatment using repeated T2 * (the decay of transverse magnetization seen with gradient-echo sequences) mapping, which is followed by histopathological correlations of MRI findings.

Results have shown stronger hypointense (dark) nanoparticle enhancement of intratibial osteosarcomas after treatment with CD47 mAb compared with IgG1 control antibody. The DT2 * enhancement, quantified as the difference between pre- and post-contrast T2 *-values, was significantly higher in CD47 mAb-treated tumors compared to IgG1-treated tumors (*p* = 0.03). In addition, the tumor DT2 * enhancement positively correlated with the quantity of CD80 and inducible nitric oxide synthase (iNOS)-positive TAM on the histology and the tumor size on post-treatment scans. Reproducibility studies are ongoing to determine variables that might affect our quantitative measures. Preliminary results demonstrated less than 10% inter- and intra-individual variations in quantitative MRI measures of TAM. Important variables that affected quantitative T2 * mapping results included magnetic field strength, tumor type, tumor size, and nanoparticle type, as well as pulse sequence parameters and the approach for image analysis. Statistical analyses of these variables are ongoing.

Meanwhile, the team started imaging studies of patients enrolled in a clinical trial for the evaluation of the efficacy of CD47 mAb. The team obtained MRI scans before CD47 mAb therapy, including a precontrast MRI scan on Day 1, intravenous infusion of ferumoxytol at a dose of 5 mg Fe/kg, and a postcontrast scan at 24 h after intravenous ferumoxytol infusion, using T2 *-weighted MR images. Before nanoparticle infusion, the tumor tissue was hyperintense compared to adjacent muscle. After the intravenous infusion of ferumoxytol, the tumor tissue became hypointense (dark), as expected based on results from our preclinical studies. While the accrual of patients is ongoing, the imaging findings will be correlated with the degree of tumor necrosis after tumor resection.

Next, the team developed a web-based server application with detailed records of both individual images and experimental data relevant to our project, which includes a web-based 3D/4D image viewer to easily and quickly view and evaluate images. The application uses a data-driven, bottom-up approach to record each piece of data and its relationship to specific experiments, studies, and projects. The original unstructured data collected from various imaging modalities and related animal preparation experiments are annotated and stored in a structured hierarchical database. Data can be managed, accessed, and shared through either private or public web clients for better data security and accessibility. The application also includes a robust image viewer and modular processing tools for various computationally intensive and artificial intelligence (AI) applications. A centralized distributed data storage is used for accessibility, scalability, security, and performance. The application provides easy access to send images to a publicly accessible image repository portal. Using this web portal, data sharing can be performed easily, provided the receiver has authorized login credentials for the application. Users can choose to send their private images to this image gallery for public viewing. Images in the public portal can be searched for using search parameters, such as user info and date of acquisition, and by selected keywords. Data sharing is fully controlled and protected by credentials accessible only to authorized users. An integrated image viewer can be used to visualize one or more selected images. The viewer features an easily navigable set of standard medical image viewing tools, such as annotation and segmentation tools. The viewer is capable of loading and overlaying multiple images in both 3D and 4D image formats, making it easy to view and compare multiple related images simultaneously.

This quantitative imaging (QI) tool for TAM imaging is readily clinically translatable and will provide important quantitative information about tumor response to cancer immunotherapy, which will inform personalized treatment protocols. Therefore, this new QI tool test will have significant impact on clinical outcomes and enable broad applications beyond the team’s immediate research focus.

### 2.5. Penn Quantitative MRI Resource for Pancreatic Cancer

**Figure 5:**
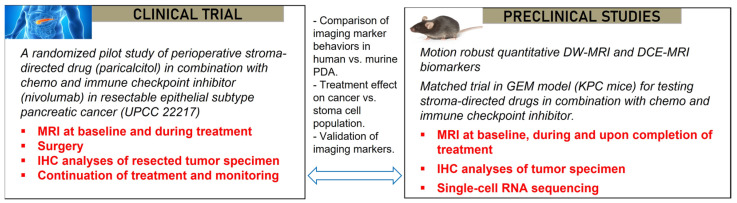
Co-clinical trial design for assessing MRI markers of tumor microenvironment changes in pancreatic cancer in response to a stroma-directed drug combined with chemo and immune checkpoint inhibitor.

The co-clinical trial of the University of Pennsylvania focuses on pancreatic ductal adenocarcinoma (PDA), employs a GEMM in the preclinical arm, and enrolls resectable PDA patients in the clinical trial (ClinicalTrials.gov ID: NCT03519308) (Figure 5). The dense extracellular matrix (stroma) in PDA harbors a unique tumor microenvironment (TME) that is immune suppressive and underlies a resistance of PDA to chemotherapy. Hence, the treatment tested in the co-clinical trial includes a stromal-directed agent (vitamin D receptor ligand) because earlier studies have shown that the activation of vitamin D receptors on stromal cells using synthetic vitamin D such as Paricalcitol reprograms the dense stroma in PDA, leading to decreased fibrosis, increased delivery of gemcitabine, and delayed tumor growth in animal models [52]. The primary goal of the co-clinical trial is to test the utility of diffusion-weighted (DW)-MRI- and dynamic contrast-enhanced (DCE)-MRI-derived QI markers for detecting the effect of a stromal-directed agent combined with chemotherapy on tumor progression and TME features such as microvascular perfusion and the extent of fibrosis (e.g., collagen deposition). One important aspect of the co-clinical trial is to develop motion-robust DW- and DCE-MRI methods since DW-MRI of mouse abdomen is challenged by the corruption and artefacts induced by the high rate of respiratory motion, which also causes blurs and suboptimal image quality in DCE-MRI of abdominal cancers.

Patients with resectable PDA are randomized into one of two arms: chemoimmunotherapy (gemcitabine, cisplatin, nab-paclitaxel, and nivolumab), or chemoimmunotherapy plus vitamin D receptor ligand, Paricalcitol. After two cycles of treatments, patients undergo surgical resection of PDA and tumor specimens are examined by IHC. DW-MRI and DCE-MRI are performed at baseline and after one cycle of treatment.

In GEMM, the initiation and progression of PDA are driven by pancreatic epithelial-specific mutations of the KRAS oncogene and the TP53 tumor-suppressor gene (Kras^G12D^:Trp53^R172H^:Pdx1-Cre), referred to as KPC, and resemble key features of human PDA including a dense stroma [53,54]. KPC mice w bred at the Mouse Hospital of the Abramson Cancer Center, University of Pennsylvania. KPC mice spontaneously developed premalignant Pancreatic Intraepithelial Neoplastic (“PanIN”) lesions at 7–10 weeks of age, leading to invasive PDAC at 17–19 weeks with high penetrance. Tumor screening was undertaken via weekly abdominal palpations starting at 11 weeks of age, followed by ultrasound examination to estimate the tumor sizes. KPC mice (both sexes, 18–25 weeks old) with tumors of sizes in the range of 70–130 mm^3^ confirmed by MRI were enrolled in the treatment study. 

KPC mice are randomized and enrolled in one of four groups: (1) control (saline); (2) chemotherapy only; (3) chemotherapy + Calcipotriol (a synthetic vitamin D for mice); (4) chemotherapy + Calcipotriol + PD-L1 mAb, where chemotherapy is a combination of three agents (gemcitabine/cisplatin/nab-paclitaxel administered at dose of 266/8/4 mg/kg), PD-L1 antibody (200 µg/mouse), and Calcipotriol (60 µg/kg). Treatment and MRI study last for 2 weeks for the preclinical trial arm. Chemotherapeutic drugs are administered on Day 0 and Day 7; calcipotriol on Monday to Friday skipping Saturday and Sunday; and PD-L1 mAb twice a week. T_2_W, DW-, and DCE-MRI are applied on Day 0 (baseline) and Day 7 followed by scheduled drug treatments, and finally, T_2_W and DW-MRI are applied on Day 14 followed by euthanasia and the harvesting of tumor tissues for IHC and for single cell RNA-seq.

**Figure 6:**
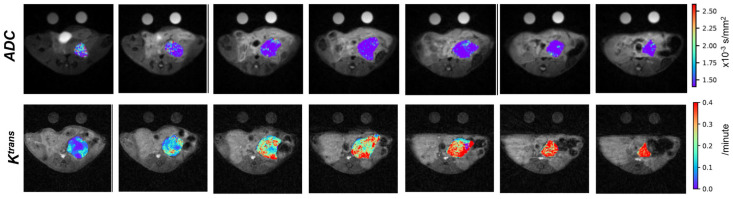
ADC maps (**upper** row) and Ktrans maps (**bottom** row) of PDA tumor from a KPC mouse. Parametric maps are overlaid on T2W images and displayed in pseudo color using the color bars on the right. Suitable phantoms for DW- and DCE-MRI are scanned with the mouse for quality control.

Quantitative imaging biomarkers (QIB) including tumor size, apparent diffusion coefficient (ADC), K^trans^, V_e_, and T_1_ relaxation time of the tumor are obtained for the co-clinical trial. We developed motion-resistant radial k-space sampling acquisition and image reconstruction protocols [55,56]. Further optimization of these protocols has led to respiratory motion-free DW and DCE images acquired from free-breathing mice without the need for respiration gating [56,57]; a deep-learning method to accelerate the DW-MRI acquisition is being developed. Tumor ADC and K^trans^ maps are shown in Figure 6 and capture the spatial heterogeneities of these features. With optimized protocols, we found that the ADC and Kurtosis index, both derived from DW-MRI, can differentiate between IPMN (intraductal papillary mucinous neoplasm) versus PDA, represented by respective GEMM [57].

Strengths of the co-clinical trial design include: (1) the neoadjuvant setting of the clinical trial allows for mechanistic insights of the treatment by detailed IHC analyses; (2) GEMM captures the stromal and other key features of human PDA; (3) there are closely matched clinical and preclinical trial designs in treatment and endpoints, while animal studies include more groups to dissect the effect of chemo, immune and stromal treatment; and (4) the co-clinical trial allows for the assessment of fibrosis and tumor immune microenvironment in both human subjects and in KPC mice. Weaknesses include the fact that resectable PDA accounts for only 20% of all PDA, and subtyping a biopsied tumor is required before enrollment, limiting the sample size of the clinical trial and impacting the statistical power.

### 2.6. University of Michigan Quantitative Bone Marrow MRI in Myelofibrosis

The University of Michigan co-clinical study centers on myelofibrosis (MF), a chronic, ultimately fatal hematologic cancer that arises either as a primary malignancy or secondary to other rare myeloproliferative neoplasms (MPNs), polycythemia vera (PV), or essential thrombocythemia (ET). Major driver mutations causing MPNs constitutively activate JAK2 kinase signaling in hematopoietic stem and progenitor cells (HSCs). Patients with primary or secondary MF commonly develop debilitating systemic inflammatory symptoms, progressive fibrosis of bone marrow and other organs, and clonal proliferation of HSCs in organs outside of bone marrow. These hallmark phenotypes distinguish MF from other MPNs. Treatment options for MF remain very limited. The U.S. Food and Drug Administration (FDA) has approved only three drugs for MF, which alleviate symptoms in ~50% of patients but do not eliminate malignant HSCs or reverse major organ pathologies. Most of these patients discontinue treatment within three years because of side effects or drug resistance.

Biopsy remains the current standard for analyzing bone marrow in patients. This technique has notable limitations, including sampling only a very small amount of bone marrow from one site (iliac crest) in a disease known to exhibit marked heterogeneity within bone marrow. In patients with advanced MF, biopsy may yield no diagnostic information, inflicting the pain of the procedure on a patient without any benefit. Imaging in clinical trials for MF has been restricted to quantifying changes in spleen volume, with a 35% reduction after six months of treatment being the standard for FDA approval of a therapy. Imaging has not been part of clinical management of patients with MF. The objective of this research is to develop quantitative imaging methods which can be validated for use in analyzing bone marrow pathology in MF, the major site of disease, in mouse models and human participants undergoing therapy. If the establishment of validated quantitative imaging biomarkers is successful, we anticipate the establishment of a new paradigm for staging MF patients and monitoring the response to therapy. Employing quantitative MRI methods for the detection of stabilization or reversion of bone marrow toward a healthy state would provide a new and important approach for clinical patient management.

The clinical study at the University of Michigan (ClinicalTrials.gov ID: NCT01973881) is currently open and enrolling participants with MF who are beginning treatment with a new drug. Patients typically undergo DNA sequencing for somatic mutations associated with myelofibrosis. There is no repeat sequencing after therapy. Studies to date have focused on patients beginning treatment with the JAK2 inhibitor ruxolitinib dosed according to the clinical judgement of the oncologist. MR imaging studies are conducted within one month of therapy initiation and follow-up scans are acquired after 1, 3, and 6 months of treatment. If a participant remains on the same therapy, they may opt to have additional imaging studies at 12 and 24 months of treatment. For all imaging studies, measurement of spleen volume is accomplished by anatomic imaging and bone marrow is monitored using the following MRI parameters in the lumbar spine, pelvis, and proximal femurs: (1) ADC for changes in cellularity; (2) magnetization transfer ratio (MTR) for extracellular macromolecules such as reticulin and collagen fibrosis; and (3) proton density fat fraction (PDFF) for amounts of fat and water (cells) content in the bone marrow. MTR images are only acquired in the pelvis because of imaging time and energy (radiofrequency deposition) constraints. MRI parameters are quantified to determine site-specific anatomic changes over the course of therapy. In addition, imaging data are also evaluated for correlation with multiple clinical metrics, including complete blood counts, biopsy, clinical symptom score according to the Dynamic International Prognostic Scoring System (DIPSS), and somatic mutations identified by targeted genomic sequencing. These data will enable the determination of the extent of response to therapy that quantitative bone marrow MRI metrics detect and the evaluation of discordance between effects of therapy on spleen volume versus bone marrow changes.

**Figure 7:**
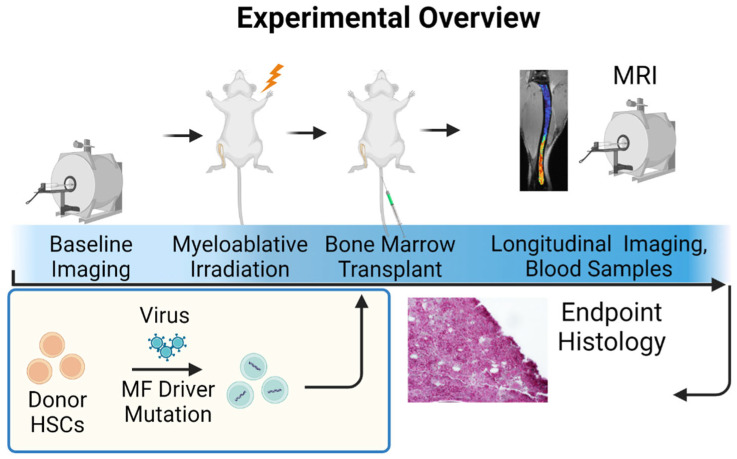
Bone marrow transplant model for myelofibrosis in mice. Baseline spleen and bone marrow MRI data are obtained prior to transplantation. To generate mice with myelofibrosis (MF), normal donor hematopoietic stem and progenitor cells (HSCs) are transduced with a known driver mutation. Transduced HSCs are transplanted into recipient mice after myeloablative irradiation, using bone marrow MRI and spleen volume to quantify disease progression and response to therapy. The same MRI methods are used for clinical studies in patients with MF.

Mouse models of MF consist of a bone marrow transplantation model to establish MF in immunocompetent mice (Figure 7). In this model, HSCs from donor mice are transduced with retroviruses expressing one of three major driver mutations for MF. Alternatively, HSCs from mice genetically modified to encode a driver mutation for MF are also available. Transplantation of HSCs into recipient Balb/c mice is accomplished, which reconstitutes the bone marrow of sub-lethally irradiated mice and then proliferates to produce characteristic disease phenotypes of MF. This co-clinical trial uses the same imaging and data analysis methods as the clinical study, although imaging of mouse bone marrow in mice is focused only on the tibia due to the use of an rf cryo-probe to improve the signal-to-noise ratio and resolution [58]. Mice have haematopoietically active bone marrow throughout the appendicular skeleton, so this approach images bone marrow that changes dynamically during disease progression and therapy. As an example, baseline images are obtained prior to the initiation of therapy with ruxolitinib, which is administered twice daily by oral gavage [59]. Imaging of mice continues at approximately 7–10-day intervals throughout therapy. Intervals between imaging studies are shorter because of the accelerated time course of disease progression in the bone marrow transplantation model of MF. The mouse model allows for testing and optimization of MRI methods for translation to human studies. The availability of mouse histology sections from the entire tibia of mice also allows for validation of imaging findings at desired time points, enabling validation of defined MRI metrics. Our work to date shows a high (0.9 or greater) correlation between MRI metrics and histologic features of the disease, establishing that MRI can detect the extent, magnitude, and heterogeneity of bone marrow abnormalities in MF, including treatment reversal. Our group has also validated the reproducibility of MRI metrics through test-retest studies. Overall, the ability to integrate quantitative MRI biomarkers into treatment studies in our mouse model of MF provides for a unique, clinically translatable approach to testing established and new therapeutic interventions for improving the outcomes of patients with MF.

### 2.7. Washington University St. Louis Co-Clinical Quantitative Imaging of Breast Cancer to Predict Response to Therapy

Washington University (WU) School of Medicine in St. Louis has two projects focused on predicting response to therapy in breast cancer (BCa) under the umbrella of the Washington University Co-Clinical Imaging Research Resource (WU-C2IR2). The first project focused on predicting response to carboplatin/docetaxel therapy in triple-negative breast cancer (TNBC), while the new project is focused on predicting response to endocrine therapy (ET) in advanced estrogen receptor-positive (ER+) BCa. The resource generates PDX matched to the patient’s tumor subtype and PDX generated from patient-specific tumor biopsies/engraftments. The use of PDX enables bi-directional translation as a test-bed for both imaging and therapeutic strategies, mindful of the limitations of PDX as summarized previously [1]. QI algorithms are harmonized, optimized, and validated in both settings and implemented in the co-clinical imaging trial to assess/predict response to therapy in BCa. Genoproteomic discovery follows both the clinical and preclinical protocols to correlate imaging biomarkers to genomic and proteomic biomarkers.

**Figure 8:**
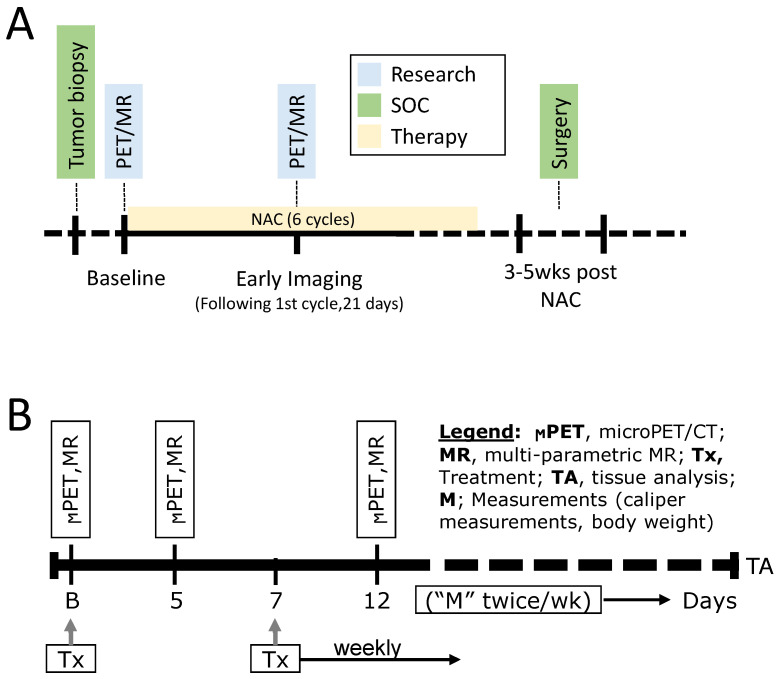
(**A**) Clinical imaging protocol and (**B**) preclinical imaging protocol to assess response to carboplatin/docetaxel therapy in TNBC.

TNBC is a highly heterogeneous and aggressive tumor characterized by poor outcome and higher relapse rates compared to other subtypes of BCa. Pathologic complete response (pCR) is often used as an important endpoint in the treatment of TNBC following neoadjuvant chemotherapy (NAC) as it is often associated with favorable long-term outcome. Therefore, it is critical to identify patients who will respond to NAC, and thus avoid the use of ineffective treatments in nonresponding patients with an opportunity to devise adaptive treatment strategies. Patients with newly diagnosed clinical stage II or III BCa with complete surgical excision of the cancer after NAC were recruited to the now completed clinical trial (ClinicalTrials.gov ID: NCT02124902). In clinical studies, docetaxel was administered intravenously at a dose of 75 mg/m^2^ over 60 min on Day 1 of each 21-day cycle. Carboplatin AUC 6 was administered intravenously over 30 min on Day 1 of each 21-day cycle immediately following docetaxel infusion. A total of six cycles were given. In preclinical studies, PDX were treated with combined docetaxel (20 mg/kg) and carboplatin (50 mg/kg) weekly for six weeks, during which period the tumor growth profile was monitored. A multi-parametric PET/MR imaging protocol including FDG-PET, T_1_w–T_2_w, DWI- and DCE-MRI was implemented in the clinical setting to predict response to therapy. Subjects were imaged at baseline (B) and again at the conclusion of the first cycle of NAC, and before starting the second cycle (Figure 8A). The preclinical imaging protocol was similar to the clinical imaging protocol, in that PDX were imaged on Days 5 and 12 post-baseline imaging (Figure 8B). We generated a panel of six PDX based on the TNBC PAM50 subtyping [60] matching patients’ tumor subtypes. These six TNBC PDX subtypes were used as a platform to optimize quantitative preclinical imaging pipelines. Thus far, we have optimized FDG-PET quantification in PDX to predict response to therapy in the co-clinical trial; characterized the impact of diet/animal stress on FDG-PET QI metrics in PDX [61]; optimized the location of orthotopic PDX tumor in the fourth mammary fat pad and developed a 1-h multi-parametric MRI protocol to suppress respiratory motion [62]; assessed and harmonized the sensitivity of radiomic features to tumor volume, image noise and resolution in preclinical PDX and clinical T_1_-weighted and T_2_-weighted MR imaging [63]; developed and validated an automated segmentation pipeline of PDX tumors in MR imaging for high throughput QI analytics [64]; optimized a FDG-PET radiomic signature to predict response to therapy in PDX; and implemented the optimized signature across the co-clinical trial to predict response to therapy [65].

**Figure 9:**
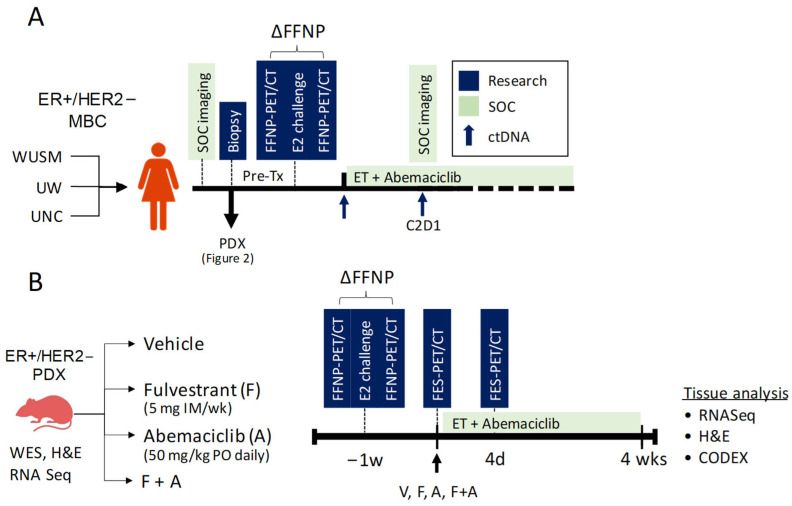
Co-clinical study design. (**A**) Multicenter clinical imaging trial to predict response to ET using ΔFFNP-PET in patients with advanced ER+/HER- BCa. Tissue biopsies will be used for genopro-teomic discovery and to generate PDX. (**B**) The preclinical PDX imaging protocol to assess the efficacy ΔFFNP-PET and FES-PET in predicting response to Fulvestrant and Abemaciclib, alone and in combination, followed by pathology and genoproteomic discovery.

As noted above, the new project is focused on predicting response to ET in advanced ER+ BCa. Approximately 70% of BCa are ER+ and human epidermal growth factor receptor 2-negative (HER2-) [66]. ET reduces recurrence risk and improves survival for many in this group. However, despite standard of care (SoC) adjuvant ET, over 20% of patients with ER+/HER2-BCa experience metastatic recurrence in the years to come, and virtually all patients with metastatic disease eventually experience disease progression on ET due to intrinsic or acquired resistance mechanisms. There are currently no biomarkers that reliably identify which of these advanced BCa patients will benefit from these ET-based approaches so that chemotherapy could be avoided or delayed. The progesterone receptor (PgR) gene is highly regulated by ER at the RNA and protein level, and thus expression of PgR in ER+ BCa would be indicative of the functional status of ER and the associated predictive benefit from ET. In the new project, we will develop co-clinical quantitative PET/CT imaging strategies to predict response to ET in patients with ER+/HER2− metastatic BCa. We will optimize, validate, and implement ^18^F-fluoroestradiol (FES)-PET and ^18^F-fluorofuranylnorprogesterone (FFNP)-PET QI strategies to assess the heterogeneity of hormone receptor status as predictors of response to ET in a panel of subtype-matched PDX to assess and compare the efficacy of FES-PET- and FFNP-PET-optimized QI metrics to predict response to therapy, and to correlate with mutation status and gene signatures of ER and PgR response to therapy. The co-clinical trial will interface with a recently funded phase II multicenter translational BCa research consortium (TBCRC) trial (PI: Dr. Farrokh Dehdashti; Co-PI: Dr. Hannah Linden) sponsored by the breast cancer research foundation (BCRF) to assess the accuracy of FFNP-PET in predicting response to abemaciclib, a CDK4/6i, plus ET in patients with ER+/HER2- metastatic BCa (Figure 9A). Patients will receive abemaciclib at a dose of 150 mg by mouth twice daily throughout every 28-day cycle. In the preclinical setting, PDX will be randomized to receive vehicle or study drug therapy with fulvestrant [5 mg intramuscular (IM) × 1 each week] or abemaciclib (50 mg/kg PO daily) or the combination of fulvestrant and abemaciclib (Figure 9B). Biopsies provided through multicenter trial and tumors from the PDX will provide high-value, multi-scale analytic data, including whole exome sequencing (WES), RNA Seq, spatial transcriptomics, circulating tumor (ctDNA), pathology, and multifunctional CODetection by indEXing (CODEX) to integrate with QI and to provide mechanistic insight into differences in response to therapy.

Overall, these projects aim to have high, far-reaching impact on the implementation of co-clinical imaging strategies in identifying, stratifying, and predicting response to therapy in TNBC and ER+/HER2 − BCa, integrating QI with genoproteomic discovery. In addition, high-value, multi-scale analytic data will be generated to interrogate and characterize tumor heterogeneity. All publications, data, and protocols are available through the WU-C2IR2 Resource website at https://c2ir2.wustl.edu (accessed on 20 January 2023).

### 2.8. Baylor/UT Austin/Stanford University Integration of Omics and Quantitative Imaging Data in Co-Clinical Trials to Predict Treatment Response in Triple-Negative Breast Cancer

The collaborative team at Baylor College of Medicine (BCM)/UT Austin/Stanford University is building MIRACCL (Molecular and Imaging Response Analysis of Co-Clinical Trials; https://miraccl.research.bcm.edu/miraccl), a web-based resource to store, manage, analyze, and display comparative imaging and omics analyses in co-clinical trials. MIRACCL integrates three existing web platforms: (1) the BCM PDX Portal (https://pdxportal.research.bcm.edu/pdxportal/), (2) ePad [67], and (3) Linked-omics [68]. The ultimate intention is to use MIRACCL to analyze and visualize data from a co-clinical trial which will evaluate four cycles of combination carboplatin/paclitaxel in both a patient cohort and a biosimilar PDX cohort, with MRI prior to, after the first cycle of, and after the completion of treatment (if medically necessary). The clinical trial has not yet been initiated, but the PDX studies have begun.

TNBC PDX models [69] are chosen based on previously determined resistance or sensitivity to combination docetaxel and carboplatin treatment [70]. Fresh PDX tumor tissues are transplanted into the cleared #4 fat pad (right inguinal) of four-week-old SCID/beige (C.B-17/IcrHsd-Prkdc^scid^Lyst^bg-J^) mice [71]. On average, it takes 2–6 weeks to detect tumors after tissue fragment implantation. Across all models, it can take anywhere from 4–10 weeks before the average tumor size of the cohort reaches the appropriate volume to initiate the study (175 mm^3^). Once tumors are visible and/or palpable, caliper measurements are used to determine the size (LxW^2^/2).

When tumors reach an average size of ~175 mm^3^, the animals are randomized (*n* = 3) onto one-week or four-week treatment arms and pre-treatment MRI is performed (Table 2). After imaging, animals are treated intraperitoneally with 50 mg/kg carboplatin and 33 mg/kg paclitaxel. Six to seven days later, on-treatment MRI is performed on animals in the one-week treatment group. After imaging, the animals are euthanized, tumor tissue is collected for snap frozen fragments, and slices are processed for formalin-fixed paraffin-embedded (FFPE) blocks. Portions of tissue from the brain, liver, and lungs are also collected for FFPE blocks and the remaining tissue is minced and snap frozen. Whole lymph nodes and ovaries are collected for FFPE and snap frozen specimens are collected as well. The remaining three animals in the four-week treatment group are treated once a week with carboplatin and paclitaxel. One week after the fourth dose, animals receive post-treatment MRI. The animals are euthanized, and tissues collected in the same manner as the one-week cohort. WES and RNAseq are performed on the PDX and patient tumor tissue that created the PDX model, when available, to provide a baseline. WES and RNA-seq were performed on early-generation (transplant generations 2–6), untreated PDX tissue. Mutational changes have been detected in PDX models that were collected from the same patient but at different points in their treatment regimen.

From an imaging point of view, there are several issues that could potentially confound interpretation. For example, the location of the tumor in a mammary fat pad makes it susceptible to motion artifacts due to its proximity to the lung, thereby making quantitative DW-MRI and DCE-MRI more difficult. The use of axial slices (as opposed to coronal or sagittal) appears to reduce these artifacts. Additionally, this location makes identification of an arterial input function for quantitative MRI difficult, yielding data that can only be analyzed with a semi-quantitative metric (e.g., the signal enhancement ratio) or by a reference region method [72].

### 2.9. University of Washington/Fred Hutchinson Cancer Center Quantitative FDG PET Imaging of Non-Small Cell Lung Cancer in a Co-Clinical Immune Checkpoint Inhibitor Therapy Study

The University of Washington and Fred Hutchinson Cancer Center are collaborating on a co-clinical study of immune checkpoint inhibitor (ICI) therapies in non-small cell lung cancer (NSCLC). This project uses a GEMM of lung adenocarcinoma to develop, test, and implement ICI therapies of this deadly disease. The Houghton lab has developed a novel mouse model of lung cancer suitable for the study of immunotherapies by exposing LSL-Kras mutant mice to cigarette smoke (KSM) [73]. Tumors in this model harbor hundreds of single nucleotide variants (SNVs) and display the classic cigarette smoking signature highlighted by G-to-T transversions and non-synonymous to synonymous SNV ratio, such that the types of mutations to be studied here are identical to those found in humans. Carcinogen exposure, e.g., a urethane-induced tumor, does not reproduce these features, and non-smoking Kras mice possess very few SNVs and so are not sufficiently antigenic to elicit immune responses [74]. The Houghton lab has also shown that neutrophils are the most common immune cell population present in NSCLC and inversely correlate with CD8+ content [75]. More recently, the group has shown that neutrophils preclude the presence of the IFNγ signature predictive of ICI treatment success and also preclude the infiltration of CD8+ cells into the malignant portions of tumor [76]. Based on these findings, the co-clinical trial project is using the KSM mouse model to identify the mechanistic determinants of ICI treatment success vs. failure and evaluate the impact of neutrophil antagonism on ICI treatment efficacy by performing a co-clinical trial in patient and smoke-exposed mice combining a novel small molecule dual-inhibitor of CXCR1 and CXCR2 (for neutrophil depletion), with anti-PD1 antibody therapy (pembrolizumab). In addition, the Pten^fl/f^l/Lkb1^fl/fl^ (PL) mouse model of lung squamous cell carcinoma will be utilized as adenocarcinomas and squamous cell carcinomas together account for >90% of all NSCLC.

**Figure 10:**
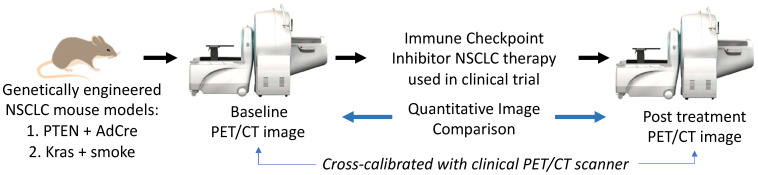
High-level schematic of co-clinical trial using PET imaging to develop image-based response criteria for preclinical studies of immune checkpoint inhibitor therapies in non-small cell lung cancer.

The scheme of the co-clinical trial is illustrated in Figure 10. Tumor formation will be initiated in KSM and PL mice via administration of intratracheal adenoviral cre (AdCre). The mice will undergo MRI every two weeks until tumors are visible, at which time PET/CT imaging will be initiated. The mice will have PET/CT scans over the timeframe in which they typically form lung tumors (20 weeks for KSM and 30 weeks for PL mice). Selected tumors will be harvested post-treatment from control and treated mice for genetic analyses. Initial efforts have centered around optimizing imaging [77] and handling protocols for reliable and reproducible results. We have encountered challenges related to the fast-growing nature of the lung tumors and drastically reduced lung capacity in the afflicted animals.

## 3. Discussion

The number of different types of animal models and their relative value for translation research in oncology have grown enormously in recent years [78,79]. Using animal models to assess therapeutic response is widespread, and the elements of experimental design needed to increase translational success have been extensively considered [80,81]. In addition, sophisticated technologies are being applied for the detection, characterization, and monitoring of cancer in animal models, generating comprehensive information about the structure, metabolism, and function of cancer cells and their microenvironment [82]. Studies focused on using animal models for imaging translation, however, have additional challenges [83]. In 2017, a consensus group assembled by Cancer Research UK and the European Organisation for Research and Treatment of Cancer published 14 key recommendations for accelerating the clinical translation of imaging biomarkers [84]. Several of these recommendations emphasized the importance of biological validation in preclinical models and the need to share, store, and curate data.

The co-clinical quantitative imaging projects of the AMCT WG of the CIRP network aim to incorporate many of these recommendations. Notable strengths among the various projects include high fidelity between tumors targeted in the clinical trial and PDX or GEMM in the co-clinical study, attempts to use human-equivalent doses of therapeutic drugs, the orthotopic location of tumors or tumors grown at cancer type-specific sites of metastases, immunocompetent hosts for murine models, and gender-appropriate murine hosts. Preclinical studies offer certain advantages compared to clinical trials, including the ability to add additional control arms, standardization of tumor volume at initiation of therapy, and more frequent imaging. Some projects take advantage of the opportunity to reverse engineer clinical acquisition and processing protocols for the optimization of associated preclinical protocols, or to optimize imaging methods preclinically for translation to human studies. The co-clinical trials aim to employ therapeutic outcomes similar to those in the clinical studies, such as tumor regression or recurrence-, disease-, and metastasis-free survival. Quantitative imaging biomarkers are sought to predict response, provide early evidence of treatment efficacy, or monitor response and/or recurrence. Several projects focus on imaging the tumor microenvironment to monitor the response of TAMs to immunotherapy or to evaluate changes in microvascular perfusion and fibrosis in response to a stromal-targeted therapy. Most projects include correlative biological studies to validate the imaging findings.

Common drawbacks among the projects include difficulty in capturing cancer heterogeneity with the small number of animal subjects in preclinical studies, the more rapid growth rate of tumors in mice compared to humans, the age of mice versus humans, and the absence of an immune microenvironment in PDX propagated in immunocompromised mice. Depending on the location of the tumors, respiratory motion impacts imaging. Means to overcome this problem include faster acquisition of images and respiratory gating.

Together with the Imaging Acquisition and Data Process (IADP) and Informatics and Outreach (IMOR) WGs, the activities of the AMCT WG contribute to the mission of the NCI CIRP to advance the practice of precision medicine by establishing consensus-based best practices for co-clinical imaging and developing optimized state-of-the-art translational quantitative imaging methodologies to enable disease detection, risk stratification, and assessment/prediction of response to therapy. The web-based resources generated by each project, including methods, protocols, software, and imaging data, will advance the efforts of the cancer research community to develop clinically translatable imaging biomarkers.

## 4. Conclusions

This article is meant to serve as a resource or handbook to investigators interested in designing co-clinical trials involving quantitative imaging. The intent is to highlight the ongoing activities of the nine teams that are part of the CIRP network and provide examples that might serve as templates for other co-clinical studies. Each section describes the associated clinical trial, the experimental approach, the rationale for the animal models selected, the imaging platforms, and pros and cons of the various elements of the co-clinical trial of each team. Work is in progress and findings will be the subject of future publications.

## Figures and Tables

**Table 1:** Overview of projects in the AMCT WG of the CIRP network.

Institution	Cancer/Disease	Model	Site	Disease Development	Therapy	Imaging
Baylor/UT Austin/Stanford	Triple-negative breast cancer	PDX	Orthotopic	2–6 weeks	Chemotherapy	mpMRI
Duke	Soft tissue sarcoma	GEMM	Orthotopic	Median 54 days	Immunotherapy Radiation Surgery	mpMRI CT
MDACC	Colorectal cancer	PDX	Subcutaneous	3 weeks	Targeted therapy	PET
Stanford	Osteosarcoma	xenografts	Orthotopic	2–3 weeks	Immunotherapy	T_2_-weighted MRI
UCSF	Small cell neuroendocrine prostate cancer	PDX	Bone, liver	1–4.5 months to reach 0.3 cc	Chemotherapy	HP MRI, mpMRI
U Mich	Myelofibrosis	GEMM	Orthotopic	21 days	Targeted therapy	mpMRI
U Penn	Pancreatic ductal adenocarcinoma	GEMM	Orthotopic	17–19 weeks	Chemoimmuno& stromal- targeted therapy	mpMRI
U Wash	Non-small cell lung cancer	GEMM	Orthotopic	20–30 weeks	Immunotherapy	PET/CT
WUSTL	Triple-negative breast cancer	PDX	Orthotopic	4 weeks–6 months	Chemotherapy	PET/MRI
WUSTL	ER+ breast cancer	PDX	Orthotopic	~3–4 months	Endocrine therapy Targeted therapy	PET

**Table 2:** Treatment schedule of preclinical trial of TNBC PDX.

Drug	Vendor	Catalog Number	Dose (mg/kg)	Concentration (mg/mL)	Vehicle	Route	Schedule
Carboplatin (Carbo)	McKesson (Teva)	740278	50	10	10 mg Mannitol per 1 mL Water	IP	Weekly
Paclitaxel (Pac)	Millipore Sigma	T7402	33	1	90% Saline/5% Kolliphor/5% Ethanol	IP	Weekly

## Data Availability

No new data were created.

## Abbreviations

adenoviral cre (AdCre); Animal Models and Co-clinical Trials (AMCT); androgen signaling inhibitors (ASI); apparent diffusion coefficient (ADC); area under the curve (AUC); artificial intelligence (AI); Baylor College of Medicine (BCM); breast cancer (BCa); breast cancer research foundation (BCRF); chemokine (C-X-C motif) ligand (CXCL); circulating tumor (ctDNA); cluster of differentiation (CD); Co-clinical Imaging Research Resource Program (CIRP); CODetection by indEXing (CODEX); colorectal cancer (CRC); computed tomography (CT); cyclin dependent kinase (CDK); diffusion-weighted (DW); dynamic contrast-enhanced (DCE); Dynamic International Prognostic Scoring System (DIPSS); endocrine therapy (ET); epidermal growth factor receptor (EGFR); essential thrombocythemia (ET); estrogen receptor-positive (ER+), fluorodeoxyglucose (FDG); fluoroestradiol (FES); fluorofuranylnorprogesterone (FFNP); Food and Drug Administration (FDA); genetically engineered mouse models (GEMM); glutaminase (GLS1); glyceraldehyde 3-phosphate dehydrogenase (GAPDH); hematopoietic stem and progenitor cells (HSCs); Human & Mouse Linked Evaluation of Tumors (HAMLET); human epidermal growth factor receptor 2-negative (HER2-), hyperpolarized (HP); Imaging Acquisition and Data Process (IADP); immune checkpoint inhibitor (ICI); immunohistochemistry (IHC); Informatics and Outreach (IMOR); inducible nitric oxide synthase (iNOS); interferon (IFN); intraductal papillary mucinous neoplasm (IPMN); intravenous (IV); janus kinase 2 (JAK2); Kras^G12D^:Trp53^R172H^:Pdx1-Cre (KPC); LSL-Kras mutant mice exposed to cigarette smoke (KSM); magnetic resonance imaging (MRI); magnetization transfer ratio (MTR); MD Anderson Cancer Center (MDACC); Mallinckrodt Institute of Radiology (MIR); metastatic castration-resistant prostate cancer (mCRPC); methylcholanthrene (MCA); Molecular and Imaging Response Analysis of Co-Clinical Trials (MIRACCL); mitogen-activated protein kinase (MAPK); monoclonal antibody (mAb), multiparametric (mp); myelofibrosis (MF); myeloproliferative neoplasms (MPNs); National Cancer Institute (NCI); neoadjuvant chemotherapy (NAC); NOD scid gamma (NSG); non-small cell lung cancer (NSCLC); orally twice a day (PO BID); pancreatic ductal adenocarcinoma (PDA); Pathologic complete response (pCR); patient-derived xenografts (PDX); PET Imaging Resource to Enhance Delivery of Individualized Cancer Therapeutics (PREDICT); pleomorphic liposarcoma (LPS); polycythemia vera (PV); positron emission tomography (PET); progesterone receptor (PgR); programmed cell death ligand 1 (PD-L1); programmed cell death protein 1 (PD-1); proton density fat fraction (PDFF); Pten^fl/f^l/Lkb1^fl/fl^ (PL), quantitative imaging (QI); quantitative imaging biomarkers (QIB); quantitative polymerase chain reaction (qPCR); radiotherapy (RT); RNA sequencing (RNA-seq); (S)-4-(3-^18^F-Fluoropropyl)-L-glutamic acid (^18^F-FSPG); signal-regulatory protein alpha (SIRPα); single nucleotide variants (SNVs); small cell neuroendocrine prostate cancer (SCNC); Spectral–Spatial (SPSP); standard of care (SoC); translational BCa research consortium (TBCRC); triple-negative breast cancer (TNBC); tumor-associated macrophages (TAM); tumor microenvironment (TME); undifferentiated pleomorphic sarcoma (UPS); University of California, San Francisco (UCSF); University of Pennsylvania (Penn); University of Texas Austin (UT Austin); whole exome sequencing (WES); aqwild-type (WT); Washington University (WU); Washington University Co-Clinical Imaging Research Resource (WU-C2IR2); working group (WG).
